# Deregulated microRNAs Are Associated with Patient Survival and Predicted to Target Genes That Modulate Lung Cancer Signaling Pathways

**DOI:** 10.3390/cancers12092711

**Published:** 2020-09-22

**Authors:** Cristiano P. Souza, Naiara C. Cinegaglia, Tainara F. Felix, Adriane F. Evangelista, Rogério A. Oliveira, Erica N. Hasimoto, Daniele C. Cataneo, Antônio J. M. Cataneo, Cristovam Scapulatempo Neto, Cristiano R. Viana, Flávia E. de Paula, Sandra A. Drigo, Robson F. Carvalho, Márcia M. C. Marques, Rui M. Reis, Patricia P. Reis

**Affiliations:** 1 Department of Surgery and Orthopedics, Faculty of Medicine, São Paulo State University, UNESP, Botucatu 18618-687, SP, Brazil; crispeixoto10@hcancerbarretos.com.br (C.P.S.); erica.hasimoto@unesp.br (E.N.H.); daniele.cataneo@unesp.br (D.C.C.); a.cataneo@unesp.br (A.J.M.C.); sandra.d.linde@unesp.br (S.A.D.); 2 Experimental Research Unity (UNIPEX), São Paulo State University, UNESP, Botucatu 18618-687, SP, Brazil; naiara.cinegaglia@unesp.br (N.C.C.); tainara.felix@unesp.br (T.F.F.); 3 Molecular Oncology Research Center, Barretos Cancer Hospital, Barretos 14784-400, SP, Brazil; adriane.evangelista@hcancerbarretos.com.br (A.F.E.); cristovamscapula@uol.com.br (C.S.N.); cristiano.viana.ext@dasa.com.br (C.R.V.); flavia.paula@hcancerbarretos.com.br (F.E.d.P.); mmcmsilveira@gmail.com (M.M.C.M.); ruireis.hcb@gmail.com (R.M.R.); 4 Department of Biostatistics, Plant Biology, Parasitology, and Zoology, Institute of Biosciences, São Paulo State University UNESP, Botucatu 18618-689, SP, Brazil; rogerio.oliveira@unesp.br; 5 Department of Structural and Functional Biology, Institute of Biosciences, São Paulo State University, UNESP, Botucatu 18618-689, SP, Brazil; robson.carvalho@unesp.br; 6 Barretos School of Health Sciences, Barretos 14785-002, SP, Brazil; 7 Life and Health Sciences Research Institute (ICVS), School of Medicine, University of Minho, 4710-057 Braga, Portugal; 8 ICVS/3B’s-PT Government Associate Laboratory, 410-057 Braga/Guimarães, Portugal

**Keywords:** lung cancer, microRNAs, target genes, pathways, survival

## Abstract

**Simple Summary:**

Lung cancer is the leading cause of cancer death, worldwide. The low survival rates are mainly due to disease diagnosis in advanced stages, and the lack of effective treatments. In this study, we analyzed molecules known as microRNAs, which regulate the expression of a large proportion of the human genes. microRNAs are involved in processes related to the development and progression of cancer. In lung cancer, many microRNAs can drive disease. This study showed that some microRNAs have aberrant levels in tumor cells of the two most common types of lung cancer: lung adenocarcinoma and squamous cell carcinoma. In addition, we found that one microRNA, named miR-25-3p, had aberrantly increased levels in tumor cells from patients who died of lung cancer. These results are useful to better understand the biology of lung cancer, and can contribute as an additional tool to predict patient outcome/survival.

**Abstract:**

(1) Background: Although the advances in diagnostic and treatment strategies, lung cancer remains the leading cause of cancer-related deaths, worldwide, with survival rates as low as 16% in developed countries. Low survival rates are mainly due to late diagnosis and the lack of effective treatment. Therefore, the identification of novel, clinically useful biomarkers is still needed for patients with advanced disease stage and poor survival. Micro(mi)RNAs are non-coding RNAs and potent regulators of gene expression with a possible role as diagnostic, prognostic and predictive biomarkers in cancer. (2) Methods: We applied global miRNA expression profiling analysis using TaqMan^®^ arrays in paired tumor and normal lung tissues (*n* = 38) from treatment-naïve patients with lung adenocarcinoma (AD; *n* = 23) and lung squamous cell carcinoma (SCC; *n* = 15). miRNA target genes were validated using The Cancer Genome Atlas (TCGA) lung AD (*n* = 561) and lung SCC (*n* = 523) RNA-Seq datasets. (3) Results: We identified 33 significantly deregulated miRNAs (fold change, FC ≥ 2.0 and *p* < 0.05) in tumors relative to normal lung tissues, regardless of tumor histology. Enrichment analysis confirmed that genes targeted by the 33 miRNAs are aberrantly expressed in lung AD and SCC, and modulate known pathways in lung cancer. Additionally, high expression of miR-25-3p was significantly associated (*p* < 0.05) with poor patient survival, when considering both tumor histologies. (4) Conclusions: miR-25-3p may be a potential prognostic biomarker in non-small cell lung cancer. Genes targeted by miRNAs regulate *EGFR* and *TGFβ* signaling, among other known pathways relevant to lung tumorigenesis.

## 1. Introduction

Lung cancer is the leading cause of cancer death worldwide. Current incidence data estimates over 2 million new cases/year, with 62% of cases occurring in developed countries, mainly in North America and Western countries, and 38% in developing countries [1]. In Brazil, incidence data estimates the occurrence of approximately 30,000 new cases/year [2]. The 5-year survival rate remains low at approximately 19%. The low survival rates are mainly due to late diagnosis, with only 15% of patients diagnosed with localized disease, 22% of patients with lymph node metastasis, 57% with distant metastasis and 6% with an undetermined disease stage [3].

Non-small cell lung cancer (NSCLC) comprises of the majority (85%) of lung cancer cases, with adenocarcinoma (AD) and squamous cell carcinoma (SCC) as the two major histological subtypes. Invasive adenocarcinoma is further classified into histological subtypes of lepidic, solid, acinar, papillary and micropapillary; the latter has been associated with a worse prognosis [4].

Global efforts have been made, in order to determine actionable mutations in NSCLC, including The Cancer Genome Atlas (TCGA) project and the Lung Cancer Mutation Consortium (LCMC) [5,6]. TCGA comprehensively mapped mutations and transcriptome changes, as well as their frequency in large sample sets of lung AD and SCC, and demonstrated that mutations in oncogenes such as *EGFR*, *K-RAS*, *ALK* and *BRAF* occur in >60% of lung AD cases [7], with driver mutations targetable by tyrosine kinase inhibitors [8]. The frequency of alterations of these driver genes in the Brazilian population is slightly different and associated with a genetic ancestry admixture [9,10,11]. Although the development of molecularly targeted therapies including EGFR tyrosine kinase inhibitors have benefited only a small fraction (15%) of patients with lung AD. The majority of patients with advanced disease are still treated with chemotherapy regimens and survival rates remain low [12]. The identification of additional genes involved in signaling pathways may be useful as novel targets for NSCLC treatment. There is still a need to identify other biomarkers able to demonstrate clinical application in patient prognosis and prediction of the treatment response [12].

In the past decade, miRNAs have been discovered as potent gene expression regulators with an important role in disease development [13]. Previous studies have shown global deregulated miRNA expression profiles in lung cancer vs. normal tissues [14]. Here, we determined the expression of a 377 miRNA panel, using the TaqMan^®^ Low Density Array platform, in primary lung AD and SCC, molecularly characterized for the major targeted genes, correlated miRNA expression with patient outcome/survival and mapped miRNA target genes in pathways associated with lung cancer. Our data adds to the current literature by showing that deregulated miRNAs modulate a number of genes encoding transcription factors, as well as common driver genes involved in lung tumor development and progression.

## 2. Results

### 2.1. Patient Clinical and Histopathological Characteristics

Of the 38 samples included in the study, 23 patients were diagnosed with lung AD and 15 with lung SCC. Based on the new histological classification of lung AD, we identified the following subtypes: acinar (*n* = 9), solid (*n* = 5), papillary (*n* = 1), lepidic with acinar component (*n* = 4) and undetermined (*n* = 4). The mean age of patients with lung AD was 60.9 years, with a similar proportion of males and females (11 and 12, respectively). About 70% of patients with lung AD were current or former smokers. Patients with lung SCC had an average age of 62 years, with a predominance of males (11 men and 4 women) and the vast majority (93.3%) were active or former smokers. Patient clinical and histopathological characteristics are summarized in Table 1.

Surgery was the primary treatment for all patients, except for one patient with stage IV disease at diagnosis, who was treated with chemotherapy. For this patient, the sample analyzed was a diagnostic biopsy obtained by lung bronchoscopy, before treatment. One patient died due to complications after surgery. Two patients developed tumor recurrence and one died of disease before palliative chemotherapy. One third of the patients received adjuvant chemotherapy (eight AD, 35% and five SCC, 33%) due to an advanced loco-regional disease (stages IB, II or III, 7th edition, Lung Cancer TNM Staging). Of these 13 patients, four developed distant metastasis requiring second line palliative chemotherapy, and two patients died due to tumor recurrence.

### 2.2. EGFR and RAS Driver Mutations in Lung AD and SCC

**Figure 1:**
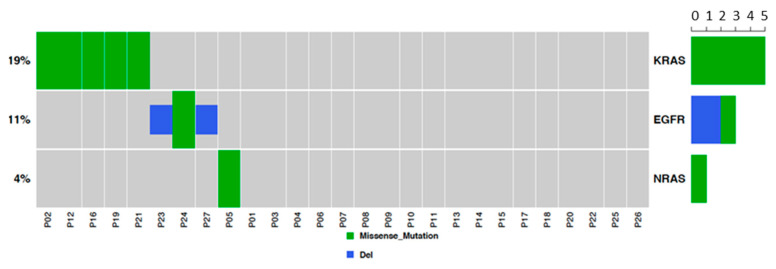
*EGFR* and *KRAS* mutation analysis. Two tumors were positive for *KRAS* p.Gly12Val, two were positive for *KRAS* p.Gly12Cys, one tumor was positive for *KRAS* p.Gly12Asp and one was positive for *NRAS* p.Gln61Arg and for *KRAS* p.Gln61His.

This analysis was performed to characterize common driver mutations (*EGFR*, *K-RAS* and *N-RAS*) in our sample set. Results showed three *EGFR* mutated tumors (13% of lung AD cases), five *K-RAS* mutated tumors (two patients; 8.7% of AD cases and three patients; 20% of SCC cases), and one *N-RAS* mutated tumor (6.7% of SCC cases; Figure 1). As expected, driver mutations were mutually exclusive. The occurrence of mutations was not associated with miRNA changes.

### 2.3. miRNAs Are Deregulated in Lung AD and SCC Compared to Normal Lung Tissues, and miR-25-3p Overexpression Is Significantly Associated with Poor Survival

Thirty-three miRNAs were detected as differentially expressed in tumors compared with normal samples, with the majority (31 miRNAs) being overexpressed (Table 2, fold change ≥ 2 and *p* < 0.05).

**Figure 2:**
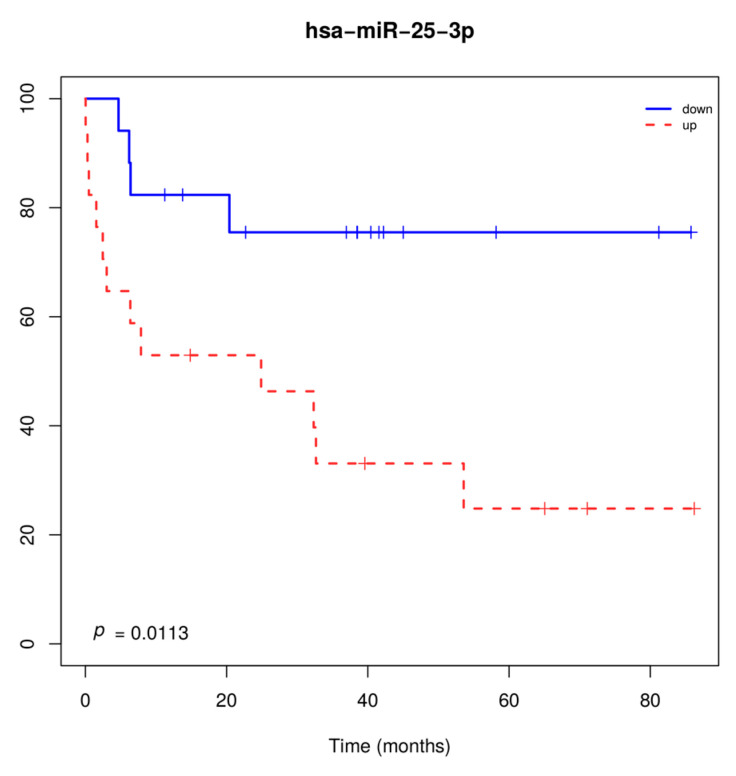
Overall survival of patients with lung carcinoma regardless of the histological subtype, according to miR-25-3p expression levels. Overexpression of miR-25-3p was associated with shorter overall survival.

Two miRNAs (miR-20a-5p and miR-93-5p) had higher expression levels in tumors from smokers compared to non-smokers (*p* < 0.05). Larger tumor size (T3-T4) and the presence of lymph node metastasis (N1-N3) were associated with higher miR-29b-3pand lower miR-95-3p expression levels, respectively. miR-25-3p overexpression was significantly associated with overall survival, when considering patients with both tumor histologies (Figure 2).

**Figure 3:**
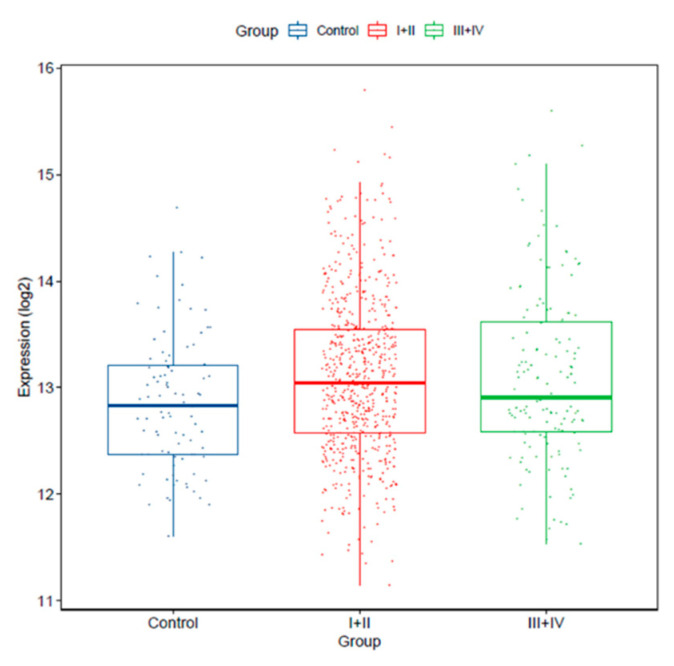
miR-25-3p expression levels is increased in stage I/II (*n* = 639; *p* = 0.01022) and III/IV (*n* = 147; *p* = 0.02359) versus normal lung tissue (control, *n* = 91; TCGA dataset).

In addition, we verified miR-25-3p expression in earlier stages (I/II; *n* = 639) and more advanced stages (III/IV; *n* = 147) tumors versus controls. We found that miR-25-3p expression was significantly increased in stage I and II tumors vs. controls (*p* = 0.01022), and in more advanced stage tumors vs. controls (*p* = 0.02359). miR-25-3p expression levels were not significantly different between tumors from stages I/II vs. III/IV (*p* = 0.4397; Figure 3).

**Figure 4:**
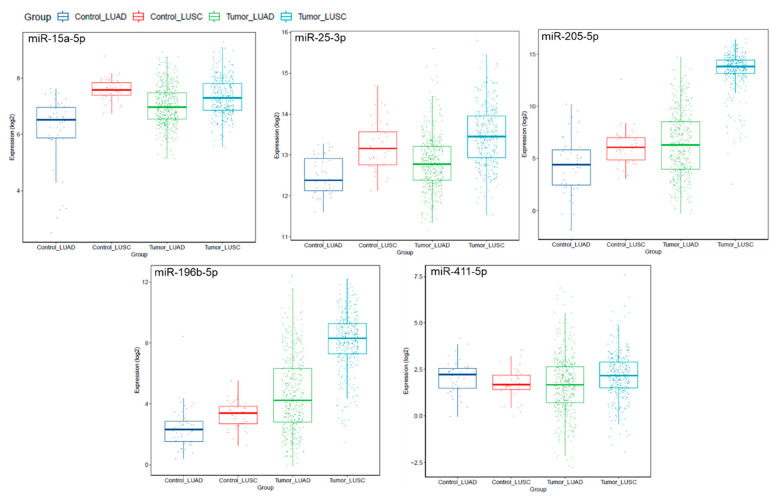
Expression levels of miRNAs miR-15a-5p, miR-25-3p, miR-205-5p, miR-196b-5p and miR-411-5p in lung adenocarcinoma (LUAD; *n* = 561) and lung squamous cell carcinoma (LUSC; *n* = 523), compared to normal lung tissues (control LUAD, *n* = 46; control LUSC, *n* = 45; TCGA dataset). This analysis shows miRNA expression levels by histological subtype, for better data visualization, since the TCGA data includes large sample sets.

Notably, a subset of miRNAs identified in our patient samples were aberrantly expressed in the lung AD and lung SCC-TCGA datasets, compared to paired normal lung tissues (Figure 4). Interestingly, miR-205-5p was confirmed as significantly overexpressed in our lung SCC, compared to lung AD samples (*p* = 0.0002).

### 2.4. miRNAs Are Predicted to Regulate Genes Abnormally Expressed in Lung AD and SCC, which Modulate Known Pathways of Lung Cancer

**Figure 5:**
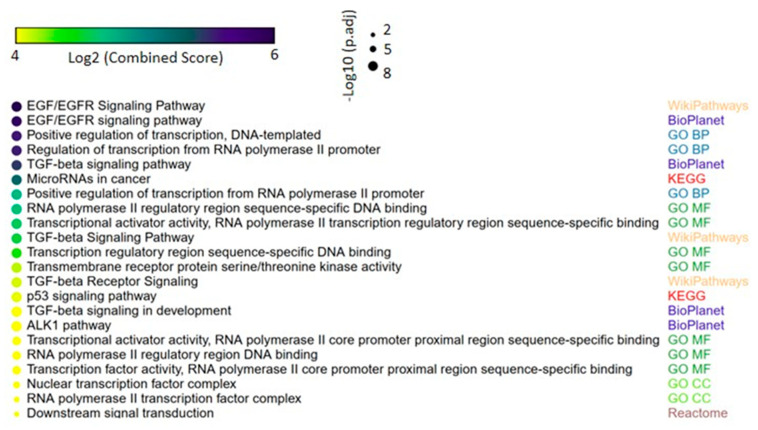
Gene set enrichment analysis of the predicted miRNA-target genes in lung cancer. Up to four of most significant terms from each of the following collections are shown: Kyoto Encyclopedia of Genes and Genomes (KEGG), WikiPathways, BioPlanet, Reactome, Gene Ontology (GO) biological process, GO molecular function and GO cellular component. The size and the color in the heat-scatterplot represent the combined score and the corresponding adjusted *p*-values, respectively, from the enrichment significance of each gene set, as computed by EnrichR [15,16].

We next evaluated the target genes potentially regulated by the 33 miRNAs identified in lung carcinomas. This analysis revealed various target genes involved in important biological functions in tumorigenesis such as transcriptional control. Interestingly, miRNAs validated in the TCGA dataset (miR-15a-5p, miR-25-3p, miR-205-5p, miR-196b-5p and miR-411-5p) were related to genes in cancer pathways. Considering all of the 33 deregulated miRNAs, significantly enriched pathways included *EGFR* and *TGFβ* signal transduction, which are known to be involved in lung cancer development and progression (Figure 5). Table S1 shows the complete list of enriched pathways, associated *p*-values and genes in each pathway.

## 3. Discussion

miRNAs are gene expression regulators with a relevant role in tumorigenesis, including lung cancer [14]. miRNA alterations often lead to target gene deregulation and deregulated signaling pathways with roles in tumor development and progression [17,18,19]. Here, we reported 33 significantly deregulated miRNAs with the vast majority (31 miRNAs) being overexpressed in lung AD and SCC compared to paired normal lung tissues. A previous study by our group, conducted in a geographically distinct subset of patients, showed that circulating miRNAs in plasma from patients with lung AD and SCC have a predominant miRNA overexpression pattern. Interestingly, among miRNAs composing three plasma miRNA signatures, miR-155-5p overexpression was a common finding [20]. Notably, in a third, independent Brazilian patient lung adenocarcinoma subset, from our group, miR-155 and miR-21 were commonly overexpressed miRNAs in a paired analysis, in tumors compared to the normal tissue from the same patients [21].

In our patient dataset, higher miR-25-3p expression levels were positively correlated with overall patient survival (*p* < 0.05) including both tumor histologies. Deregulated miR-25 expression has been reported in human cancer [22,23], and overexpressed in NSCLC primary tumors and cell lines, associated with increased cell proliferation, migration and invasion [24]. miR-25 was further shown as overexpressed in NSCLC compared with paired normal lung tissues, and demonstrated to activate ERK signaling via *KLF4*, a miR-25 target gene, leading to increased tumor cell migration and invasion [25]. miR-25 overexpression was also associated with tumor progression and prognosis in lung adenocarcinoma [26], and shown to modulate radiation-induced apoptosis in radiotherapy-resistant tumors [27]. Conversely, downregulated miR-25 inhibited cell proliferation, induced G1 cell cycle arrest, increased cisplatin sensitivity and suppressed growth of cancer cell xenografts [28]. Additionally, high levels of miR-25 were detected in serum from NSCLC patients, associated with adverse prognostic factors such as tumor stage and lymph node metastasis, as well as overall survival and recurrence-free survival [29]. These data, along with our results, strongly suggest that miR-25 has a prognostic role in lung carcinoma.

In the TCGA NSCLC cohort, miR-25-3p overexpression was not associated with survival (*p* = 0.2), likely due to overrepresentation of earlier stage (I/II) lung AD and SCC tumors, which are often associated with better survival. In addition, while the TCGA dataset largely contributes to the validation of molecular changes associated with tumorigenesis mechanisms, previous reports suggested that uncurated data in large datasets might lead to biased correlation of biomarkers with clinical outcomes [30,31].

Other overexpressed miRNAs detected here, such as miR-196b-5p, were previously reported in lung cancer [32], and suggested to have a prognostic value in gastric cancer and glioblastoma [33,34]. Among the molecular targets of miR-196, Annexin 1 expression was associated with increased cell invasion [35], suggesting an oncogenic role in NSCLC. miR-196b upregulation was also demonstrated to promote cell invasion and morphological cellular alterations [36,37]. In addition, miR-205 has been identified by others [32,38,39,40,41] and suggested as a circulating biomarker with a diagnostic value in NSCLC [42]. Experimental evidence indicate that miR-205 acts in lung tumorigenesis through *PTEN* inactivation, promoting growth, migration, invasion and chemoresistance in NSCLC [43].

A limitation of our study is the small number of tumors, and a limited miRNA panel included in the analyses. In order to overcome this limitation, we included external miRNA expression validation in the large lung AD and SCC publicly available RNA-Seq datasets from TCGA.

Based on TCGA data, pathways activated by *EGFR* mutations were found in 11% of tumors, *K-RAS* mutations in 32%, *PI3K* mutations in 4%, *HER2* in 3% and *PTEN* inactivation in 3% of lung adenocarcinoma samples [6]. In lung SCC, TCGA data showed that pathways are often activated by mutations in *TP53* in 81% of tumors, *PI3K* in 16%, *PTEN* in 15%, *EGFR* in 9%, *KRAS* in 3% and *HER2* in 4% of tumors [5]. Although we did not find mutations associated with miRNA changes, likely due to our small sample set, we identified common, mutually exclusive driver mutations in *EGFR*, *K-RAS* and *N-RAS*. In Brazilian patients, mutational frequency of driver genes was reported in a large number of lung adenocarcinoma cases (*n* = 444), with *EGFR* mutated in 22.7% and *K-RAS* in 20.4% of the cases [9].

By computational analyses, we showed that deregulated miRNAs modulate a number of genes encoding transcription factors, as well as common driver genes in lung cancer. Enrichment analysis showed signal transduction pathways including *EGFR*, *TGFβ* and *PI3K-AKT*, which are implicated in lung tumor development and progression [44].

## 4. Material and Methods

### 4.1. Ethics Statement

This study was performed in accordance with the Declaration of Helsinki and national and international ethics guidelines. Our study has been approved by the Research Ethics Boards of the Faculty of Medicine, UNESP, Botucatu, SP (4319/2012) and Barretos Cancer Hospital, Barretos, SP (75907).

### 4.2. Patient Samples

Fifty-eight formalin-fixed, paraffin-embedded (FFPE) tissue blocks were obtained from patients diagnosed with lung adenocarcinoma and squamous cell carcinoma. Patients have undergone surgery as the primary treatment, from 2007 to 2012, in two centers in the state of São Paulo; Botucatu Clinical Hospital and Barretos Cancer Hospital, SP, Brazil. All FFPE tissue samples were subjected to RNA and DNA extraction, as outlined below. Of these, 19 samples were excluded due to low RNA concentration. An additional nine histologically normal lung tissues, adjacent to their corresponding tumors, were collected and used as reference samples. Clinical and histopathological characteristics were obtained from medical records including age at diagnosis, gender, date of surgery, history of tobacco and alcohol exposure, disease grade and stage, type of primary treatment and/or adjuvant and/or palliative treatment, last date of follow-up and outcome. These data are shown in Table 1.

### 4.3. RNA and DNA Extraction

Samples obtained from FFPE tissue blocks were subjected to RNA extraction using the RecoverAll Total Nucleic Acid Isolation kit (Ambion/Life Technologies, Carlsbad, CA, USA), following a previously reported protocol with modifications to improve RNA yield [45]. RNA samples were quantified using NanoDrop 8000 (Thermo Fisher Scientific, Waltham, MA, USA) and quality was assessed using Bionalyzer 2100 (Agilent Technologies, Santa Clara, CA, USA), following the manufacturer’s protocol. RNA samples were immediately stored at −80 °C until use for miRNA expression analysis.

In addition, DNA was isolated from representative sections of tumor FFPE resected samples, using the QIAmp DNA micro kit (Qiagen, Hilden, Germany) following the manufacturer’s instructions as previously reported [9]. DNA was used for analysis of driver mutations by the SNaPShot assay.

### 4.4. SNaPShot Assay

Mutational analysis was performed using previously described primers without the MET primer, which was removed from the assay [45]. The PCR protocol was adapted as below, and performed in a final volume of 10µL, with 50 ng of DNA and 1µM of forward and reverse primers, using 5 µL of the HotStar master mix multiplex (Qiagen, Hilden, Germany) according to the manufacturer´s protocol, with the cycling parameters: 95 °C for 15 min, followed by 40 cycles of 95 °C for 30 s, 60 °C for 1 min. 30 s., 72 °C for 1 min and 72 °C for 30 min in a thermal cycler (Veriti, Applied Biosystems, Carlsbad, CA, USA). PCR products were purified with EXO-SAP (Affymetrix, Santa Clara, CA, USA) for 1 h for 37 °C and 15 min for 80 °C. The SNaPShot assay was performed as multiplex extension reactions using 5µL of the reaction mix (SNapShot, Applied Biosystems) following the manufacturer´s protocol, with the cycling parameters: 96 °C for 30 s, followed by 35 cycles of 96 °C for 10 s, 50 °C for 5 s, 60 °C for 30 s and 60 °C for 10 min carried out in a thermal cycler (Veriti, Applied Biosystems, Carlsbad, CA, USA). The extension products were separated by electrophoresis in the ABI 3500 XL genetic analyzer (Applied Biosystems/Thermo Fisher Scientific); data were analyzed using the ABI GeneMapper, version 4.0 software (Applied Biosystems/Thermo Fisher Scientific).

### 4.5. EGFR Exon 19 Deletion Analysis

EGFR exon 19 deletions were evaluated using previously described primers [45]. The PCR assay was performed in a final volume of 15 µL, with 50ng of genomic DNA and 10µM of forward and reverse primers, using 7.5 µL of the HotStar master mix (Qiagen, Hilden, Germany) following the manufacturer´s protocol, with the cycling parameters: 96 °C for 15 min, followed by 40 cycles of 96 °C for 45 s, 56.5 °C for 45 s, 72 °C for 45 s and 72 °C for 10 min carried out in a thermal cycler (Veriti, Applied Biosystems, Carlsbad, CA, USA). The PCR products were separated by electrophoresis in the ABI3500 XL genetic analyzer (Applied Biosystems/Thermo Fisher Scientific) and data were analyzed using the ABI GeneMapper v.4.0 software (Applied Biosystems/Thermo Fisher Scientific).

### 4.6. EGFR Exon 20 Insertion Analysis

A mutation analysis was performed using previously described primers [45]. The PCR assay was performed in a final volume of 15 µL, with 50ng of DNA and 10µM of forward and reverse primers, using 7.5 µL of the HotStar master mix (Qiagen, Hilden, Germany) following the protocol proposed by the manufacturer, with the cycling parameters: 96 °C for 15 min, followed by 40 cycles of 96 °C for 45 s, 56.5 °C for 45 s, 72 °C for 45 s and 72 °C for 10 min were carried out in a thermal cycler (Veriti, Applied Biosystems, Carlsbad, USA). PCR products were purified with EXOSAP (Affymetrix, Santa Clara, CA, USA) and subjected to direct sequencing using a BigDye Terminator cycle sequencing and BigDye X Terminator purification kit (Applied Biosystems). The analysis was performed with the software Genetic Analyzer ABI PRISM 3500 and SeqScape version 2.7 (Applied Biosystems).

### 4.7. Quantitative miRNA Expression Analysis by TaqMan Low Density Arrays

miRNA expression was assessed by using the TaqMan^®^ Array Human MicroRNA platform, card A, containing 377 miRNAs (Life Technologies, Foster City, CA, USA), as previously described [46]. Briefly, the reverse transcription mix was prepared using Megaplex primers, followed by preparation of the quantitative PCR mix (450 µL of amplification mix plus 6 µL of newly synthesized cDNA, added to 444 µL of nuclease-free ddH2O (Sigma, St. Louis, MI, USA)). Of this mix 100 µL was added to the cards containing lyophilized miRNA probes; the cards were then centrifuged and loaded into the QuantStudio 12K instrument (Life Technologies, Foster City, CA, USA). miRNA expression profiles were determined in a total of 41 samples (38 tumors and 3 pools of 9 histologically normal lung tissues from a subset of the patients. Each pooled control contained three normal lung samples. Pooled samples were prepared after RNA extraction from each sample. Global normalization was performed using the Expression Suite software (Life Technologies, Foster City, CA, USA) using the stably expressed endogenous controls RNU-44, RNU-48 and U6, and compared to pools of normal lung samples.miRNA expression profiles were determined using the RQ Manager v.1.2 software (Life Technologies, Foster City, CA, USA), and the Delta Delta Ct method [47].

### 4.8. Computational and Statistical Analyses

Data from the TCGA dataset was retrieved using FirebrowseR [48]. Predicted miRNA target genes were identified using miRWalk v. 3.0 (http://mirwalk.umm.uni-heidelberg.de/) [49] by integrating the prediction results of TargetScan [50], miRDB [51] and miRTarBase [52], and considering a score ≥ 0.95 as the screening threshold. The predicted targets were used to perform a comprehensive gene set enrichment analysis with EnrichR links (http://amp.pharm.mssm.edu/Enrichr/) [15,16,53]. The enrichment results of the top ten terms from each EnrichR link were represented by the *p*-value (Fisher’s exact test) and Z score (correction to the test) in a combined score [15,16]. A heat scatterplot for the enrichment results was created using the web tool Morpheus [54] (https://software.broadinstitute.org/morpheus) [55].

## 5. Conclusions

miR-25-3p overexpression may have prognostic relevance in lung AD and SCC. In addition, our data support the existing literature by identifying miRNAs predicted to modulate transcription factors and known driver genes with a role in lung cancer pathways. Our data show that different miRNAs are biologically relevant in NSCLC.

## Figures and Tables

**Table 1:** Demographic and clinicopathological data of patients with lung adenocarcinoma (AD) and squamous cell carcinoma (SCC).

Variables	AD	SCC	*p*
Age			
Mean (SD)	60.91 (10.3)	62 (7.1)	0.725
Range	40–84	51–73	
Sex	N (%)	N (%)	
Male	13 (56.5)	11 (73.4)	0.329
Female	10 (43.5)	4 (26.6)	
Smoking	N (%)	N (%)	
No	7 (30.5)	1 (6.7)	0.114
Yes	16 (69.5)	14 (93.3)	
Stage	N (%)	N (%)	
I	9 (39.1)	4 (26.7)	0.651
II	6 (26.1)	4 (26.7)	
III	8 (34.8)	6 (40)	
IV	0	1 (6.6)	
Death	*n* (%)	*n* (%)	
Cancer-associated	5 (62.5)	4 (44.4)	0.637
Other causes	3 (37.5)	8 (56.4)	

*n*: number. Variables are not statistically significantly different between patients with lung AD and SCC.

**Table 2:** Significantly deregulated miRNAs in NSCLC, sorted based on fold change (FC) values, from lower to higher.

miRNA	FC	*p* Value of FC
miR-143-3p	0.326	<0.001
miR-140-5p	0.369	0.049
miR-376c-3p	2.057	0.001
miR-141-3p	2.060	0.004
**miR-20a-5p**	2.107	0.003
miR-199a-3p	2.199	0.003
miR-374-5p	2.260	<0.001
miR-130a-3p	2.414	< 0.001
**miR-29b-3p**	2.511	<0.001
let-7d-5p	2.580	<0.001
**miR-93-5p**	2.751	<0.001
miR-142-3p	2.920	0.007
miR-15a-5p	3.016	0.017
miR-155-5p	3.056	0.005
miR-25-3p *	3.371	<0.001
miR-429	3.593	<0.001
miR-452-5p	3.668	0.007
miR-20b-5p	3.917	<0.001
miR-135b-5p	4.026	<0.001
miR-708-5p	4.354	<0.001
miR-200b-3p	4.446	<0.001
miR-340-5p	5.345	<0.001
miR-744-5p	5.649	<0.001
miR-365-3p	5.766	<0.001
miR-205-5p	5.856	0.001
miR-590-5p	6.112	0.001
miR-224-5p	6.692	<0.001
miR-15b-5p	6.712	<0.001
miR-21-5p	7.827	<0.001
**miR-95-3p**	9.817	<0.001
miR-31-5p	13.929	0.001
miR-196b-5p	16.525	0.001
miR-411-5p	25.909	0.033

FC: fold change. Bolded miRNAs were associated with clinical data. * indicates the miRNA correlated with lower overall survival.

## Supplementary Materials

The following are available online at https://www.mdpi.com/2072-6694/12/9/2711/s1, Table S1: List of enriched pathways, associated *p*-values and genes in each pathway.
